# Supplementary material for: Aptamer-Based Recognition of Breast Tumor Cells: A New Era for Breast Cancer Diagnosis
Source: Int J Mol Sci. 2024 Jan 10;25(2):840. doi: 10.3390/ijms25020840 (PMC10815801; doi:10.3390/ijms25020840)
Supplement: Supplementary file 1 [file ijms-25-00840-s001.zip › ijms-2674455-supplementary.pdf]

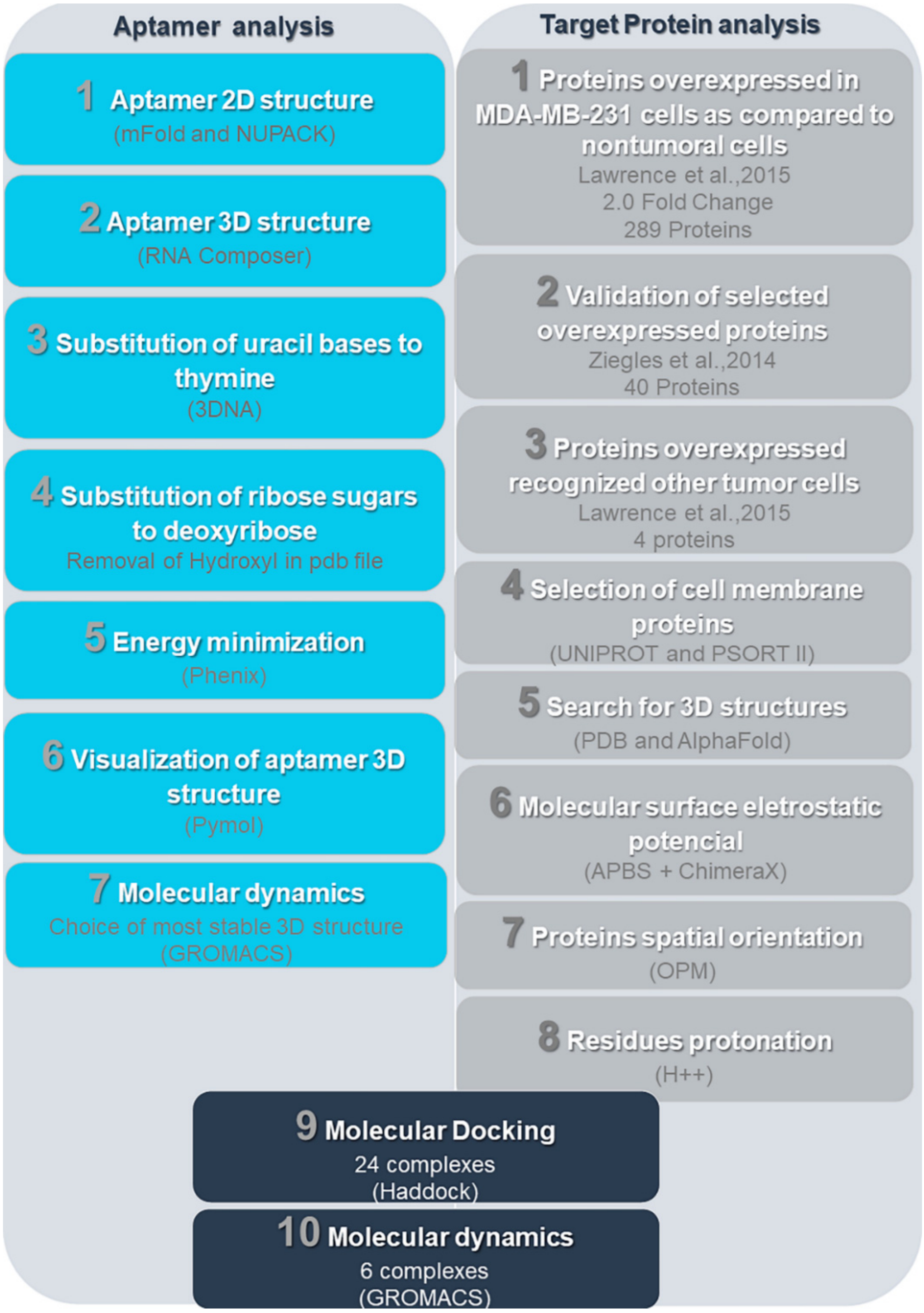

Figure S1. Workflow for predicting the structure of aptamers and selecting target proteins.

Table S1. Plasma membrane proteins differentially expressed in the MDA-MB-231 strain selected after the validation stage.

Gene	ID UNIPROT	Cellular location (Uniprot)
**CSKP**	O14936-3	plasma membrane
**TMEM205**	K7ELQ9	plasma membrane
**TM209**	Q96SK2-2	plasma membrane
**TM245**	Q9H330-2	plasma membrane
**SCAM4**	Q969E2-2	plasma membrane
**AGRE5**	P48960	plasma membrane

**G3V2K7**	G3V2K7	plasma membrane
**TM9S3**	Q9HD45	plasma membrane
**AT2B1**	P20020-6	plasma membrane
**AT2B3**	Q16720-8	plasma membrane
**E9PL82**	E9PL82	plasma membrane
**1A03HLA-A**	P04439	plasma membrane
**TSP1**	P07996	plasma membrane
**CD151**	P48509	plasma membrane
**VAMP7**	P51809	plasma membrane
**ADAM9**	Q13443	plasma membrane
**E41L2**	O43491	plasma membrane
**E9PPD9**	E9PPD9	plasma membrane
**TMX4**	A2BDY9	plasma membrane
**CD63**	F8VV56	plasma membrane
**HLA-A**	P10316	plasma membrane
**EGFR**	P00533	plasma membrane
**PIP4P2**	D6RBT7	plasma membrane
**TMCC1**	O94876	plasma membrane
**TM55B**	U6CTL2	plasma membrane
**CD44**	P16070-4	plasma membrane
**EPHA2**	P29317	plasma membrane
**NCS1**	P62166	plasma membrane
**TMEM106C**	Q9NUM4	plasma membrane
**CEMIP2**	Q9UHN6	plasma membrane
**MAGI3**	Q5TCQ9	plasma membrane
**TMEM237**	Q96Q45	plasma membrane
**TMEM181**	Q9P2C4	plasma membrane
**VAPA**	Q9P0L0	plasma membrane
**TMED10**	G3V2K7	plasma membrane
**TMCO4**	Q5TGY1	plasma membrane
**CD46**	Q6VE48	plasma membrane
**SMAP1**	Q8IYB5	plasma membrane
**TMEM199**	Q8N511	plasma membrane
**TMTC3**	Q6ZXV5	plasma membrane

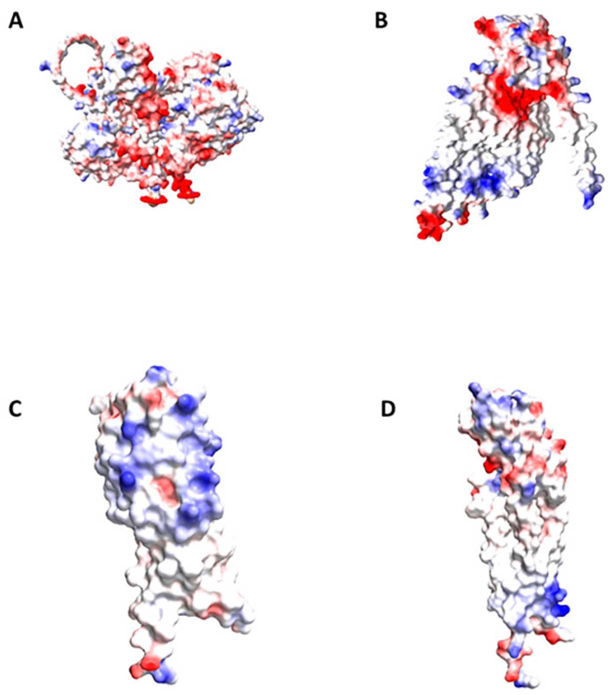

Figure S2. Representation of the electrostatic potential of the proteins selected for the molecular docking step. Analysis of the electrostatic potential of the CASPK (**A**), TM9S3 (**B**), TMEM205 (**C**), and CD151 (**D**) proteins. Blue indicates a positive charge and red indicates a negative charge.
